# Case of simultaneous occurrence of hepatitis, cholangitis, and pancreatitis as immune-related adverse events induced by immune checkpoint inhibitor therapy: a case report

**DOI:** 10.1007/s00261-025-04994-w

**Published:** 2025-05-27

**Authors:** Kana Kawata, Dai Inoue, Takahiro Komori, Takashi Matsubara, Fumihito Toshima, Kazuto Kozaka, Masahiro Yanagi, Hiroko Ikeda, Satoshi Kobayashi

**Affiliations:** Kanazawa University Hospital, Kanazawa, Japan

**Keywords:** Immune checkpoint inhibitors, Adverse events, Hepatitis, Cholangitis, Pancreatitis, CT

## Abstract

The use of immune checkpoint inhibitors has increased in the field of oncology; however, various immune-related adverse events affecting multiple organs have been reported. Herein, we present a case of concurrent hepatitis, cholangitis, and pancreatitis as immune-related adverse events (irAE); a case of autoimmune disease due to oncologic immunotherapy. A man in his 80s who was undergoing pembrolizumab therapy for recurrent renal pelvic cancer presented to the emergency department with a loss of appetite. Laboratory tests revealed elevated levels of inflammatory markers and liver enzymes. Initial non-contrast computed tomography (CT) suggested cholecystitis and cholangitis, for which antibiotics were administered. However, because of poor improvement, contrast-enhanced dynamic CT and gadolinium-ethoxybenzyl-diethylenetriamine-pentaacetic acid-enhanced magnetic resonance imaging (MRI) were performed two weeks after visiting the emergency department to reassess the underlying cause. In these examinations, besides the bile dust wall thickening and edematous changes along Glisson’s sheath suggesting the cholangitis, inflammatory enlargement in pancreatic tail was also revealed. Considering these imaging findings suggesting the cholangitis and pancreatitis during pembrolizumab therapy, irAE was suspected as the cause of symptoms. A liver biopsy subsequently performed strongly indicated hepatitis and cholangitis as irAE. Based on these findings, concurrent hepatitis, cholangitis, and pancreatitis as irAE by pembrolizumab were diagnosed. Imaging findings of irAE cholangitis are similar to those of primary sclerosing cholangitis and IgG4-related cholangitis. Particularly in cases like this one, where pancreatitis is also present. However, if a history of immune checkpoint inhibitor use is known, it is possible to include irAE in the differential diagnosis, as observed in this case. Therefore, by keeping the use of immune checkpoint inhibitors in mind during imaging interpretation, imaging examinations could be a clue to suggest the possibility of irAE. Recognizing the imaging findings associated with irAEs and the existence of cases where irAE cholangitis and irAE pancreatitis coexist, it can aid earlier diagnosis of irAEs.

## Introduction

Immune checkpoint inhibitors (ICIs) have been widely approved as standard treatments for malignancies, with expanding indications in oncology. However, these drugs can cause various immune-related adverse events (irAEs) [1–4]. Common irAEs frequently encountered in clinical practice include disorders affecting the colon, liver, lungs, and endocrine system. Endocrine-related irAEs include hypopituitarism, hypothyroidism, type 1 diabetes mellitus, diabetes insipidus, and primary adrenal insufficiency [1]. So far, literatures about irAE pancreatitis, hepatitis and cholangitis have been reported, however, concurrent occurrence of these conditions has not been published [1–4].

We encountered a case of simultaneous onset of the irAEs hepatitis, cholangitis, and pancreatitis during ICI therapy. Liver injury associated with ICIs, particularly PD-1 inhibitors, has been reported in approximately 4-10% of cases [5], while irAE cholangitis has an incidence of up to 3% [6]. Additionally, pancreatitis following ICI therapy is observed in approximately 2% of cases [7]. Additionally, hepatitis and pancreatitis as irAEs are more frequent with combination therapies involving PD-1 inhibitors and CTLA-4 inhibitors [5, 7]. To the best of our knowledge, there have been no previous reports of concurrent irAE hepatitis, cholangitis, and pancreatitis.

## Case report

A man in his 80s who had undergone pembrolizumab (PD-1 inhibitors) therapy for recurrent left renal pelvic cancer (urothelial carcinoma) after surgery presented to the emergency department approximately 7 months after initial pembrolizumab administration with a chief complaint of loss of appetite. A total dose of pembrolizumab administrated to the patient before onset was 1600 mg. Laboratory tests revealed elevated levels of inflammatory markers and liver enzymes (Table 1). Additionally, tests performed the following day showed elevated levels of pancreatic enzymes. Non-contrast computed tomography (CT) at the time of the emergency visit suggested cholecystitis and cholangitis, prompting the initiation of antibiotic therapy and discontinuation of pembrolizumab. However, owing to poor improvement in symptoms and laboratory findings, further evaluation with contrast-enhanced dynamic CT and Gadoxetic Acid-enhanced MRI was performed two weeks after the patient visited the emergency department.

**Table 1:** Laboratory data 1

Parameter	Result	Normal range
WBC	9320	3300–8600
Hb (g/dL)	6.5	13.7–16.8
Plt	36.1 × 10^4^	158–348
AST (IU/L)	942	13–33
ALT (IU/L)	535	8–42
ALP (IU/L)	2081	38–113
γ-GTP (IU/L)	942	10–47
LDH (IU/L)	370	124–222
Amy (IU/L)	528	40–113
T-bil (mg/dL)	0.5	0.3–1.2
CRP (mg/dL)	10.35	< 0.14
IgG4 (mg/dL)	81	11–121

Hb, hemoglobin; Plt, Platelets; AST, aspartate transaminase; ALT, alanine transaminase; ALP, alkaline phosphatase; γ-GTP, gamma-glutamyl; LDH, lactate dehydrogenase; Amy, amylase; T-bil, total bilirubin; BUN, blood urea nitrogen; Cr, creatinine; CRP, C-reactive protein

**Fig. 1:**
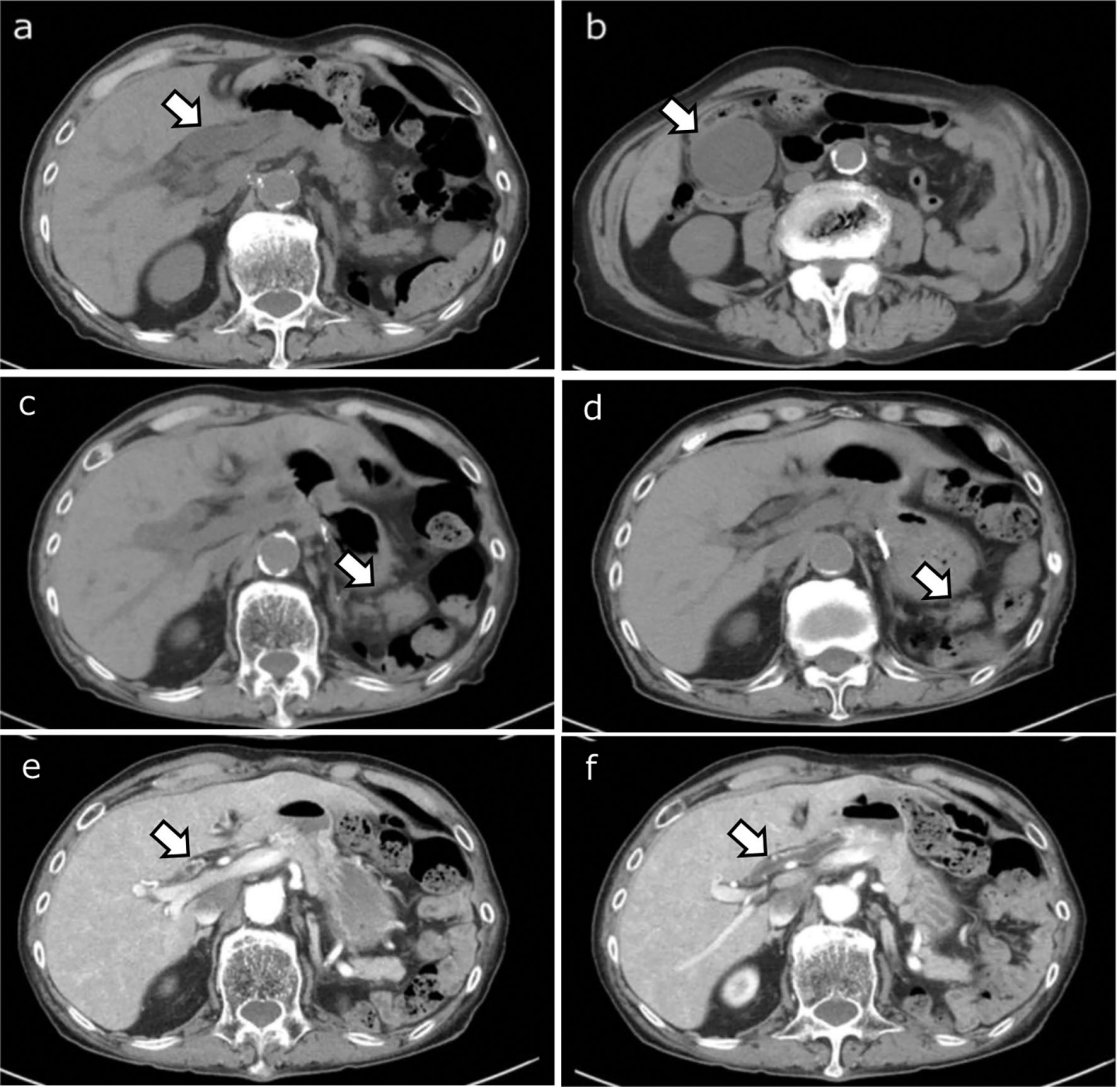
(**a**, **b**, and **c**): Non-contrast computed tomography (CT) at the time of the emergency department visit. (**d**): Non-contrast CT taken 3 months prior to the emergency department visit. (**e** and **f**): Early-phase contrast-enhanced CT (after antibiotic treatment). Mild dilatation of the common bile duct (**a**; arrow) and gallbladder enlargement (**b**; arrow) are noted. The pancreatic tail is also swollen compared to that of three months ago (**c**, **d**; arrows). Dynamic CT revealed heterogeneous enhancement in liver parenchyma (**e**) and circumferential thickening of the bile duct walls, extending from the hepatic hilum to the distal bile duct (**e**, **f**; arrows)

**Fig. 2:**
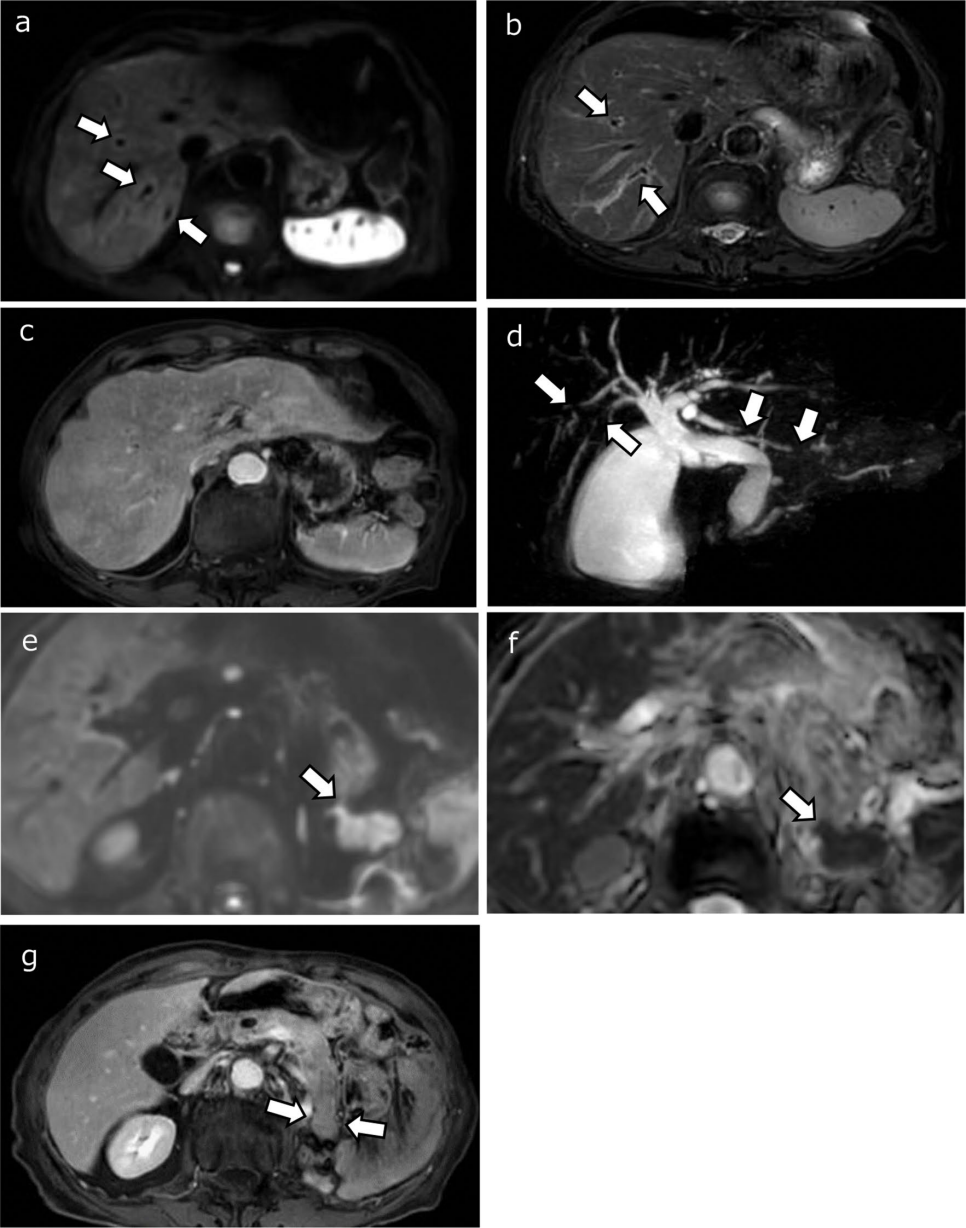
(**a** and **e**): Diffusion-weighted imaging (**b** = 800). (**b**): Fat-suppressed T2-weighted imaging. (**c**): Fat-suppressed contrast-enhanced T1-weighted imaging in the early phase (**d**): Magnetic resonance cholangiopancreatography (MRCP). (**f**): Apparent diffusion coefficient (ADC) map. (**g**): Fat-suppressed contrast-enhanced T1-weighted imaging in the portal venous phase. Faint high-signal areas are observed along Glisson’s sheath in the liver parenchyma on diffusion-weighted imaging and fat-suppressed T2-weighted imaging (**a**, **b**; arrows), suggesting edematous change with heterogeneous parenchymal enhancement in the early-phase image (**c**). Multiple luminal narrowings in the intrahepatic bile duct (**d**; arrows) and common bile duct dilatation are shown on MRCP. The enlarged pancreatic tail exhibits high signal intensity on diffusion-weighted imaging (**e**; arrow), with a reduced ADC value of 1.12 × 10^− 3^ mm^2^/s and decreased enhancement on fat-suppressed contrast-enhanced T1-weighted imaging (**f**; arrows)

In addition to gallbladder enlargement, a thickened gallbladder wall, and dilation of the common bile duct, early-phase contrast-enhanced CT showed heterogeneous enhancement of the liver parenchyma and circumferential thickening of the bile duct walls extending from the hepatic hilum to the distal bile duct (Fig. 1). MRI demonstrated hyperintensity on fat-suppressed T2-weighted imaging and diffusion-weighted imaging without apparent diffusion coefficient restriction around Glisson’s sheath, suggesting edematous changes and irregular early enhancement in the dynamic study. Mild luminal irregularities in the intrahepatic bile duct were observed on MR cholangiography. The pancreatic tail appeared enlarged compared to that in a non-contrast CT conducted 3 months earlier, showing faint hyperintensity on diffusion-weighted imaging, with a reduced ADC value of 1.12 × 10^− 3^ mm^2^/s and low signal intensity on fat-suppressed T1-weighted imaging, suggesting focal pancreatitis (Fig. 2). Pancreatic lesion showed homogeneous enhancement on dynamic study and peripancreatic inflammation was not observed. At this time, serum IgG4 levels were within the normal range. Based on the clinical course and imaging findings, irAE-related cholangitis and pancreatitis were suspected, and a liver biopsy was performed.

**Fig. 3:**
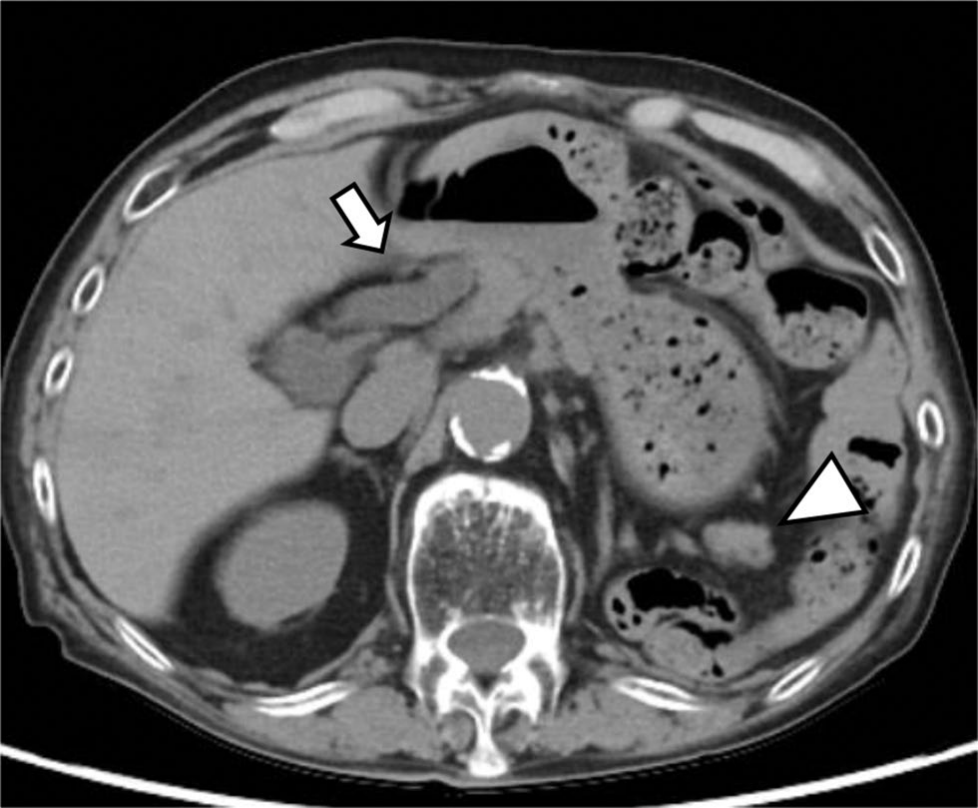
Non-contrast CT after PSL therapy. Non-contrast CT after PSL therapy shows the improvement in the bile duct wall thickening (arrow). The pancreas tail enlargement is also diminished (arrow head)

**Fig. 4:**
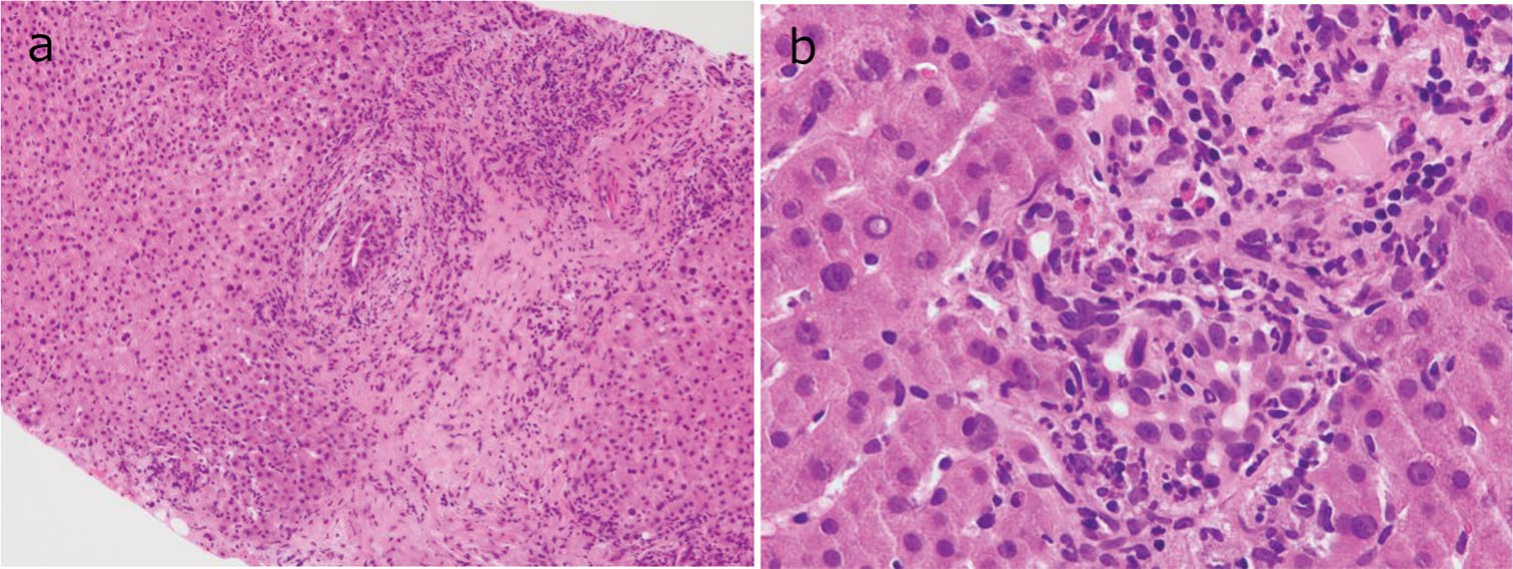
Hematoxylin and eosin staining of liver biopsy specimen (**a** and **b**). The portal areas exhibit fibrous expansion with infiltration of lymphocytes, neutrophils, and eosinophils. The interlobular bile ducts are preserved; however, the nuclei of the bile duct epithelium are enlarged, and scattered neutrophils are observed within the epithelial layers (**a**). Focal necrosis is noted in the liver parenchyma, along with zonal hepatocyte dropout in some areas (**b**)

**Fig. 5:**
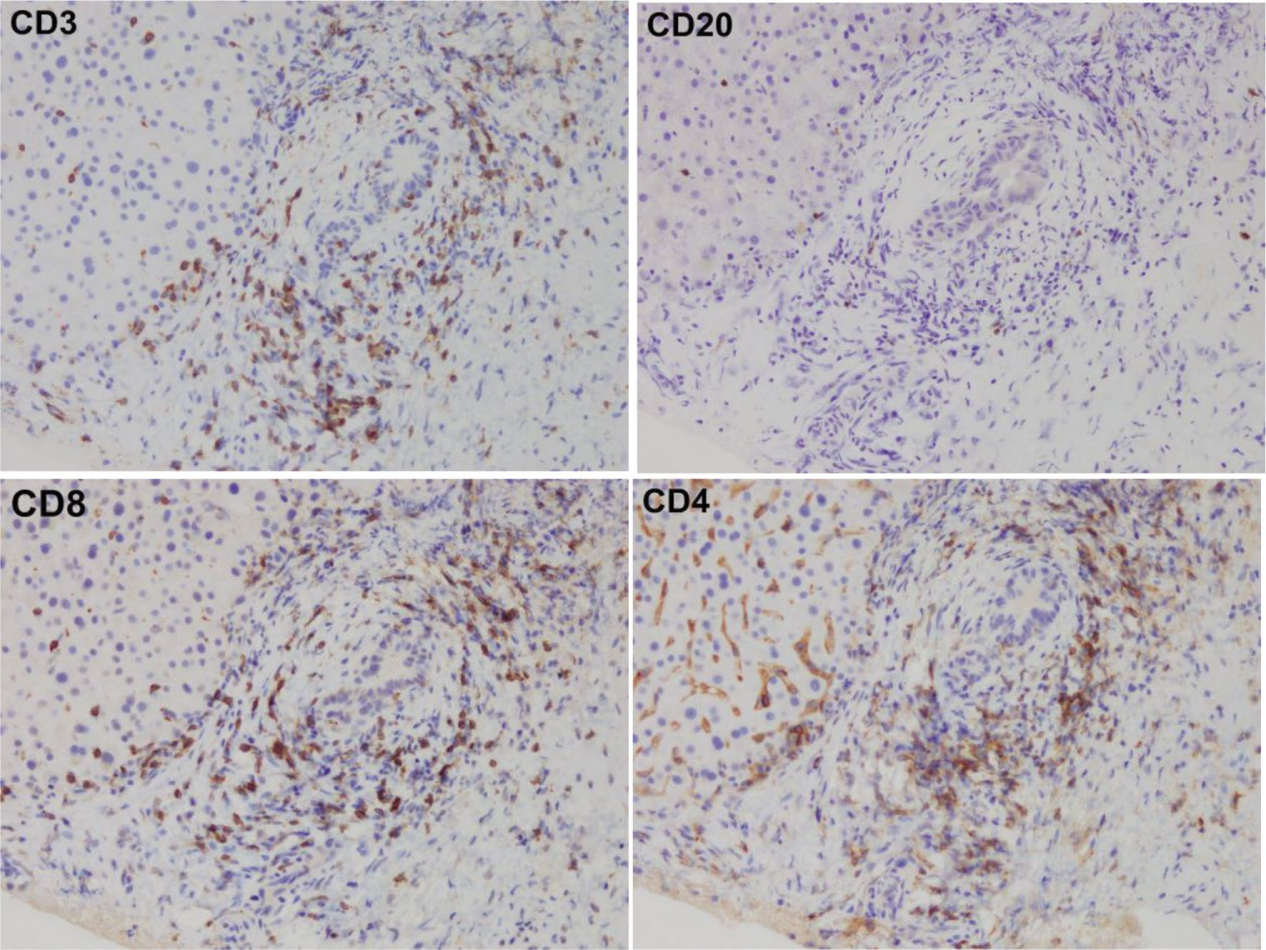
Immunohistochemical analysis revealed that the infiltrating lymphocytes were predominantly CD3(⁺) T cells, with CD4 and CD8 immunostaining showing a roughly equal distribution, though with a slight predominance of CD8

Histopathological examination with hematoxylin and eosin staining revealed fibrous expansion of the portal areas, accompanied by infiltration of lymphocytes, neutrophils, and eosinophils. The interlobular bile ducts were preserved. However, nuclear enlargement of the bile duct epithelium and scattered neutrophils were observed in the epithelial layers. Focal necrosis was observed in the liver parenchyma, and some areas showed zonal hepatocyte dropout (Fig. 3). Immunohistochemical analysis revealed that the infiltrating lymphocytes were predominantly CD3(+) T cells, with CD4 and CD8 immunostaining showing roughly equal distributions, although slightly favoring CD8 (Fig. 4). Based on these findings, a diagnosis of concurrent hepatitis, cholangitis, and pancreatitis was established. After diagnosis, the immune checkpoint inhibitor administration was stopped and prednisolone therapy was initiated at 60 mg/day, and improvements in liver and pancreatic enzyme levels were noted. Follow up plain CT obtained 2 months after starting PSL therapy also revealed minimal improvement of bile duct wall thickening and pancreas enlargement were normalized. However, due to the progression of the primary disease the patient passed away approximately nine months after the emergency department visit (Fig. 5).

## Discussion

ICIs target immune checkpoint receptors, such as CTLA-4 and PD-1, as well as the PD-1 ligand expressed on T cells. By inhibiting these pathways, ICIs enhance the immune response against tumors. However, this mechanism can also activate T cells that respond not only to tumor antigens but also to non-tumor (self-antigen) antigens, leading to irAEs [1].

Liver injury due to ICIs, particularly PD-1 inhibitors, has been reported in approximately 

4-10% of cases and is more frequent with combination therapies involving PD-1 inhibitors and CTLA-4 inhibitors. Symptoms of irAE hepatitis may include fever, jaundice, and abdominal pain; however, the condition is often asymptomatic and detected only through elevated liver enzyme levels in blood tests. Imaging abnormalities in irAE hepatitis are uncommon; however, severe cases may show hepatomegaly, heterogeneous enhancement of the liver parenchyma, periportal edema, and portal lymphadenopathy on CT or MRI [5, 8].

Recently, irAE cholangitis, a form of liver injury primarily involving the bile duct, has been reported [6]. Previously, cases of liver injury caused by ICIs, including cholangitis, were classified as irAE hepatitis. While 98% of irAE hepatitis cases resolve with or without steroid therapy, irAE cholangitis has a steroid response rate of only 11.5%, making its differentiation clinically significant [9, 10]. IrAE cholangitis occurs in up to 3% of cases and exhibits imaging features resembling sclerosing cholangitis, such as nonobstructive bile duct dilation and diffuse thickening of the extrahepatic bile duct wall [6]. Takinami et al. examined the clinical differences between irAE hepatitis and irAE cholangitis. They found that hepatitis was more common in patients treated with CTLA-4 inhibitors, whereas all cases of cholangitis occurred in those treated with PD-1 inhibitors. The median time from immunotherapy initiation to grade 2 or higher liver enzyme elevation was 55.5 d for irAE hepatitis and 257 d for irAE cholangitis. Additionally, irAE cholangitis is associated with elevated cholestatic enzyme levels, such as increased ALP levels [11]. Our case aligns with these findings, as the patient was treated with a PD-1 inhibitor and presented with cholestatic liver enzyme elevation approximately 7 months after the first dose. Reports also suggest that 13.3% of irAE cholangitis cases are accompanied by irAE hepatitis [9].

The incidence of pancreatitis following ICI therapy is approximately 2%. Similar to hepatitis, pancreatitis is often asymptomatic and is diagnosed based on elevated pancreatic enzyme levels in laboratory tests. Although there are no reports on differences in incidence rates among drug classes, combination therapy with PD-1 and CTLA-4 inhibitors increases the incidence of pancreatitis as an irAE [7]. Imaging findings on contrast-enhanced CT or MRI may include diffuse or localized pancreatic enlargement and peripancreatic edema. Patients receiving monotherapy frequently develop localized pancreatitis, whereas those undergoing combination therapy are more likely to develop diffuse pancreatitis [12]. However, pancreatic necrosis and pseudocyst formation are rare. Approximately 16% of irAE pancreatitis cases exhibit imaging findings consistent with autoimmune pancreatitis, including focal or diffuse parenchymal enlargement [12]. However, the absence of a capsule-like rim, a characteristic imaging feature of autoimmune pancreatitis, along with normal serum IgG4 levels, can aid in differentiation. Clinical information on ICI use is crucial. After treatment, 44% of patients with irAE pancreatitis develop pancreatic atrophy, and 36% develop exocrine or endocrine pancreatic insufficiency, necessitating diabetes monitoring. Moderate to severe irAE pancreatitis responds well to steroid therapy, highlighting the importance of early diagnosis and intervention [12].

## Conclusion

To the best of our knowledge, this is the first reported case of irAEs simultaneously involving hepatitis, cholangitis, and pancreatitis. IgG4-related disease should be considered as a differential diagnosis in cases of concurrent irAE cholangitis and pancreatitis. Thus, in cases involving both cholangitis and pancreatitis, it is essential to evaluate serum IgG4 levels and assess ICI use. To date, irAE hepatitis and pancreatitis have often been reported as asymptomatic. However, further validation would be necessary in this point with large case cohorts. Anyway, imaging plays a crucial role in the diagnosis of irAEs when elevations in liver or pancreatic enzymes are observed in laboratory tests. Further investigation is warranted regarding the imaging findings of irAE-associated hepatitis, cholangitis, and pancreatitis.

## Data Availability

No datasets were generated or analysed during the current study.

## Abbreviations

MRI: Magnetic resonance imaging
CT: Computed tomography
ICI: Immune checkpoint inhibitors
irAE: Immune-related adverse events
